# Corticocortical evoked potential amplitude is altered by cortical stimulation in epilepsy

**DOI:** 10.1002/epi.70289

**Published:** 2026-05-19

**Authors:** Bethany J. Stieve, Aaron F. Struck, Dillon Scott, Richard Chappell, Andrew T. Knox

**Affiliations:** ^1^ Department of Neurology University of Wisconsin Madison Wisconsin USA; ^2^ Department of Biostatistics and Medical Informatics University of Wisconsin School of Medicine and Public Health Madison Wisconsin USA; ^3^ Present address: Department of Head and Skin Ghent University Ghent Belgium; ^4^ Present address: Department of Neurology Washington University St. Louis Missouri USA

**Keywords:** DBS, EEG biomarkers, invasive EEG, personalized medicine, RNS

## Abstract

**Objective:**

Neurostimulation is an established therapy for drug‐resistant epilepsy, but optimizing stimulation parameters remains a major challenge. Corticocortical evoked potential (CCEP) amplitude may serve as an immediate biomarker of neurostimulation effects, enabling rapid parameter optimization. This study was undertaken to determine whether changes in CCEP amplitude reflect immediate neurostimulation effects.

**Methods:**

We conducted a prospective study in 17 patients undergoing stereoelectroencephalographic monitoring. Each cortical site received randomized 5‐s stimulation trains at low (7 Hz) and high (100 Hz) frequencies, preceded and followed by 30‐s 1‐Hz trains to evoke CCEPs. We analyzed 1210 pre‐/posttreatment CCEP pairs using nonparametric tests and linear mixed‐effects modeling.

**Results:**

High‐frequency stimulation produced significantly greater reductions in CCEP amplitude than low‐frequency stimulation (median change: −1.6% vs. −.02%, *p* = .002), independent of total charge and epileptiform activity. These effects persisted for minutes to more than an hour and were most pronounced within the seizure onset zone. In contrast, low‐frequency stimulation caused greater reductions in the mesial temporal region.

**Significance:**

Relative changes in CCEP amplitude by high‐ and low‐frequency neurostimulation reflect region‐specific effects and may have utility for rapid parameter optimization. These findings highlight a potential strategy to accelerate personalization of neurostimulation therapy for epilepsy.

**Key points**

- CCEP amplitude is altered by a single 5‐s neurostimulation train.
- This effect varies with frequency and location and persists up to an hour.
- One hundred‐hertz stimulation reduces CCEP amplitude more than 7 Hz overall.
- One hundred‐hertz stimulation reduces CCEP amplitude more inside the seizure onset zone.
- Seven‐hertz stimulation reduces CCEP amplitude more inside the mesial temporal region.

## 1 INTRODUCTION

More than 1 million people in the United States live with drug‐resistant epilepsy and uncontrolled seizures that significantly decrease quality of life.^1^, ^2^ For many, neurostimulation therapies like deep brain stimulation or responsive neurostimulation (RNS) offer the best chance of seizure freedom and improved well‐being.^3^ However, 30%–90% of individuals with neurostimulation devices have ongoing seizures, highlighting the pressing need to improve device efficacy.^4^, ^5^, ^6^, ^7^, ^8^ A key limitation of device efficacy is identifying optimal stimulation parameters.^9^, ^10^, ^11^ Clinicians typically begin with generic stimulation parameters and gradually adjust them over years. As treatment response gradually improves with these adjustments,^7^, ^12^ parameter optimization emerges as a critical bottleneck for seizure freedom. Given the vast number of possible parameter combinations, reducing time required for parameter optimization remains a daunting challenge.

Algorithmic parameter optimization methods such as Bayesian optimization^13^ offer a promising solution to the challenge of selecting stimulation parameters; they have already been applied to Parkinson disease and pain management^14^, ^15^, ^16^, ^17^, ^18^, ^19^ as well as preclinical models of epilepsy.^20^, ^21^, ^22^ Although efficient, they rely on immediate, quantifiable biomarkers such as beta power^23^ or electrographic seizures^20^ to assess parameter effectiveness. In principle, Bayesian optimization could be translated directly to clinical practice; however, as seizures are relatively infrequent, alternative real‐time biomarkers are needed to make these paradigms clinically viable.

Several biomarkers of seizure propensity and neurostimulation response have been studied,^24^ including corticocortical evoked potentials (CCEPs)^25^, ^26^, ^27^ and changes in spectral power.^28^, ^29^, ^30^, ^31^ CCEPs are stimulus‐locked electroencephalographic (EEG) waveforms that reflect connectivity between the stimulus and response regions.^32^, ^33^, ^34^, ^35^ Mechanistically, CCEPs represent propagating waves of neuronal depolarization elicited by direct current stimulation; consequently, their amplitude may reflect the excitability of associated cortex. A potential real‐time biomarker to be utilized in data‐driven parameter optimization paradigms could be immediately measured, would be differently modulated by stimulation, and should have a plausible link to clinical outcomes. CCEP amplitude meets these criteria; it has been previously demonstrated to vary with stimulation features such as charge^36^, ^37^ and has been used (directly or indirectly) to identify the seizure onset zone (SOZ)^25^, ^38^, ^39^, ^40^, ^41^, ^42^ and assess optimal locations for long‐term neurostimulation in patients with invasive EEG monitoring.^40^, ^42^, ^43^, ^44^ Furthermore, measuring change in CCEP amplitude removes the problem of variability in CCEP amplitude and morphology between different anatomic sites, as each site serves as its own control. However, although CCEPs have been well characterized, it is not known whether short neurostimulation trains cause a detectable change in CCEP amplitude.

To address this knowledge gap, we conducted a prospective, randomized study using single 5‐s stimulation trains, directly comparing the effect of low‐frequency stimulation (LFS; 7 Hz) and high‐frequency stimulation (HFS; 100 Hz) on CCEP amplitude. We hypothesized that CCEP amplitude would be differentially reduced by HFS and LFS, with greater effect in the SOZ.

## 2 MATERIALS AND METHODS

### 2.1 Subjects

All patients with medically refractory epilepsy undergoing intracranial stereo‐EEG (sEEG) monitoring for phase II epilepsy surgery evaluation at UW Health University Hospital or American Family Children's Hospital between August 2023 and May 2025 were invited to participate. Exclusion criteria were limited to pregnancy and incarceration. All studies were conducted in the epilepsy monitoring unit. Individuals who provided informed consent completed a single 90‐min cortical stimulation session. Intracranial EEG recordings were obtained the Natus Neuroworks/XLTEK system with a Quantum 128–256 channel base at a sampling rate of 2048 Hz (512 Hz for three individuals). Signals were amplified with the Natus Quantum LTM Amplifier, filtered between .01 Hz and 4 kHz, and stimulation was conducted with a Nicolet Cortical Stimulator (Natus). All recruitment and experimental procedures were approved by the University of Wisconsin's institutional review board.

### 2.2 Selection of stimulation sites

Anatomical targeting and placement of depth electrodes was determined by the clinical team as part of the individual's phase II epilepsy surgery workup. Between six and 14 contact cylindrical depth electrodes (90–10 platinum‐iridium; diameters of .86, 1.12, or 1.3 mm; length of 2.29, 2.41, or .8 mm; Ad‐Tech Medical Instrument Corp.) were used. Fellowship‐trained epileptologists reviewed postimplantation fused computed tomography (CT)/magnetic resonance imaging (MRI) images and sEEG recordings to select pairs of adjacent contacts on eight different electrodes that were determined to be in cortex as stimulation sites for the research protocol (Table S1). Electrodes were chosen to include both hemispheres, areas inside and outside the presumed SOZ, and areas inside and outside the mesial temporal region (MTR), resulting in a diverse set of stimulation sites across the cohort (see Figure S2).

### 2.3 Stimulation paradigm

**FIGURE 1:**
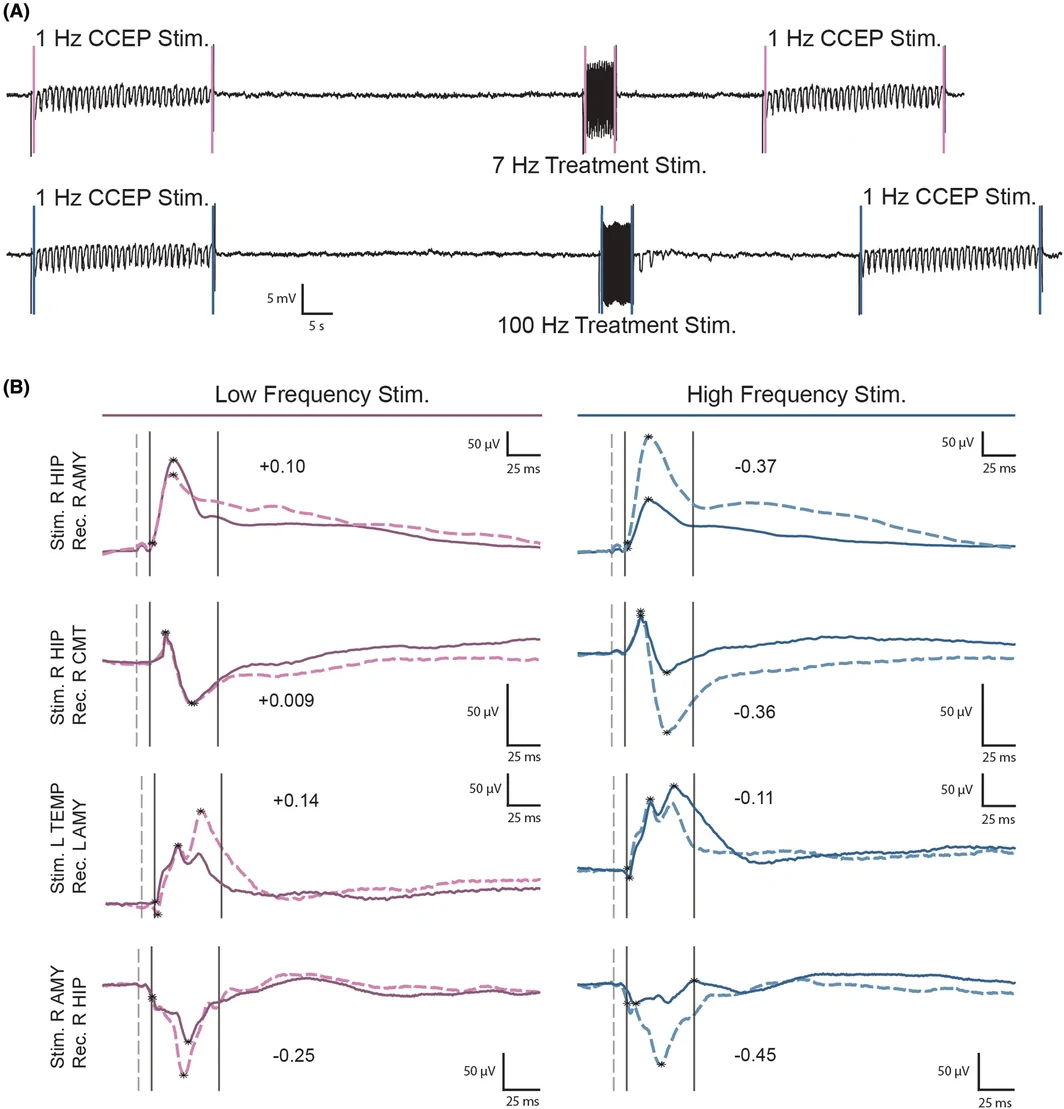
A 5‐s stimulation burst is sufficient to change corticocortical evoked potential (CCEP) amplitude. (A) Electroencephalographic traces (scale: 5 s by 5000 μV) recorded from the right amygdala during stimulation of the right hippocampus, illustrating the stimulation paradigm. At each stimulation site, CCEPs were elicited with a 30‐s 1‐Hz stimulation train. These were followed by a 5‐s “treatment” stimulation burst at low (7 Hz) or high (100 Hz) frequency, then another 30‐s CCEP stimulation train at least 30 s later. (B) Average CCEP waveforms from four different individuals (top to bottom: Subjects 5, 17, 15, and 2; scale: 25 ms by 50 μV), preceding (dashed trace) and following (solid trace) a 5‐s stimulation train at either low (left) or high (right) frequency. Each pre–post pair is annotated with the normalized change in CCEP amplitude. Asterisks show minimum and maximum values used to calculate CCEP amplitude. L TEMP: left temporal cortex; R CMT: right centromedian nucleus of the thalamus; R HIP: right hippocampus; R/L AMY: right/left amygdala; Rec., recording electrode; Stim., stimulating electrode.

We tested the effect of a 5‐s stimulation train at high (100 Hz) and low (7 Hz) frequency on CCEP amplitude given the initial treatment frequency for clinical RNS is 100 Hz and some nonresponders improve with 7‐Hz stimulation.^7^, ^12^ Each stimulation site received two runs of stimulation, each consisting of three components: (1) a 30‐s 1‐Hz stimulation train to generate baseline CCEPs, (2) a 5‐s “treatment” stimulation train at either 7 Hz or 100 Hz, and (3) a second 30‐s 1‐Hz stimulation train to generate posttreatment CCEPs (see Figure 1A).

All stimulation trains used a pulse width of 160 μs and a maximum current of 8 mA, resulting in safe maximum charge densities^45^, ^46^ between 15 and 39 μC/cm^2^. At the start of each study session (prior to evoking any CCEPs), we determined the appropriate stimulation intensity for the 5‐s train. One to three sites predicted to be prone to evoked seizures (the SOZ or MTR) were stimulated with 5‐s 100‐Hz trains, starting at .5 mA and increasing in .5–1‐mA increments up to 4 mA. The highest current that did not evoke afterdischarges was used for subsequent treatment trains.

After determining the current amplitude for the 5‐s stimulation train, we initiated the CCEP stimulation protocol. All stimulation sites were visited in a randomized order, predetermined using a random number generator. At each site, we established the CCEP train intensity by delivering 10‐s 1‐Hz trains, starting at 1 mA and increasing by 1‐mA increments up to a maximum of 8 mA. To generate CCEPs at each channel, we used the highest amplitude that did not evoke afterdischarges. Current amplitude was maximized to minimize variability of CCEP waveforms.^28^

After determining the current amplitude for CCEP stimulation, we delivered a 30‐s 1‐Hz stimulation train to evoke pretreatment CCEPs. Then, following a minimum 60‐s interval, we delivered a 5‐s treatment stimulation train. Treatment parameters (7 Hz or 100 Hz, 160‐μs pulse width) were chosen based on prior RNS applications.^7^, ^12^ During the first round of stimulation at all sites, the frequency applied was randomized to either 7 Hz or 100 Hz. At least 30 s after the treatment train ended, a second 30‐s 1‐Hz stimulation train was delivered to evoke posttreatment CCEPs. After all selected sites were stimulated, we carried out a second round of stimulation at all electrodes using the same protocol, in a new randomized order, applying the treatment stimulation frequency (100 Hz or 7 Hz) that had not been previously used. All randomization was performed in MATLAB (function: randi, shuffled seed; R2024b, MathWorks).

### 2.4 Seizure monitoring with stimulation

A fellowship‐trained epileptologist was present during all stimulation sessions and continuously monitored for seizures. Stimulation occasionally evoked afterdischarges or electrographic seizures, which were closely monitored; stimulation resumed only after epileptiform activity resolved. When feasible, the posttreatment stimulation CCEP stimulation train was delivered following electrographic or clinical seizures, although often after a delay. All instances of epileptiform activity were identified by visual review of all EEG data by a fellowship‐trained epileptologist and are listed by subject in Table S2. Following clinical seizures, participants were reminded that they could request stimulation end at any time.

### 2.5 EEG processing

We processed and analyzed EEG data using custom MATLAB scripts. Extracortical electrode contacts—identified by review of fused CT/MRI images and sEEG tracing—were removed, and channels were rereferenced to a bipolar montage. All channels were notch‐filtered at 60, 120, and 180 Hz using EEGLAB's Cleanline function.^47^ We removed stimulation artifacts by replacing data −2 to +20 ms surrounding stimulation with a reversed and interpolated EEG segment from 22 to 2 ms prior to stimulation, following a method similar to Hays et al.^31^

### 2.6 CCEP identification

We assessed all adjacent contact pairs for CCEPs, excluding the actively stimulated contacts on a given trial. In keeping with several prior studies of CCEPs,^37^, ^40^, ^41^, ^48^ EEG signals were first baseline‐corrected in the expected CCEP window (10–300 ms following each 1‐Hz stimulation pulse) by subtracting the average EEG value of a baseline period 350‐10 ms prior to that pulse. We then averaged the EEG signal from 10 to 60 ms following each of the thirty 1‐Hz stimulation pulses, the time corresponding to the N1 component of a CCEP waveform.^32^ We obtained a *Z*‐score for each channel by dividing the N1 amplitude by the SD of 30 baseline intervals; a CCEP was considered detected if the *Z*‐score exceeded 4. For each unique stimulating‐recording site pair, if a CCEP was detected during any of the four CCEP stimulation periods, CCEPs were quantified and analyzed for all other 1‐Hz stimulation trains, regardless of detection. Occasionally, stimulation artifact in the EEG signal extended beyond the offset, producing false‐positive CCEP detections due to artificially inflated baseline‐corrected signal amplitudes. To address this, we manually reviewed all detected CCEPs, and clear false positives were excluded. Across all individuals, 8% of automated detections were excluded after manual review.

### 2.7 CCEP quantification

We compared five different methods for quantifying CCEP amplitude. For the N1 component (10–60 ms poststimulation), we measured amplitude using (1) the difference between the absolute minimum and maximum of the EEG signal, (2) the root mean square of the signal, and (3) the line length^49^ of the signal over that window. For the N2 component (60–300 ms poststimulation), we used: (4) line length and (5) root mean square of the EEG signal over that window. For all methods, changes in CCEP amplitude were normalized by dividing the difference between pre‐ and posttreatment amplitudes by their sum. To analyze individual (rather than averaged) CCEP waveforms, only the absolute maximum/minimum method was used. These approaches have strengths and limitations; they may not correctly measure the amplitude of a CCEP component that spans both widows but have the advantage of detecting an effect for a variety of CCEP morphologies. Additionally, maximum/minimum and line length measures can be computed directly from data during the CCEP window and are independent of baseline correction.

### 2.8 Data modeling and statistical analysis

During the study, each selected site received four stimulation trains to evoke CCEPs—before and after both HFS and LFS treatment trains (see Figure 1A). Paired data (e.g., changes in CCEP amplitude with HFS and LFS) were compared using the Wilcoxon signed‐rank test. Differences in unpaired data were assessed using the Wilcoxon rank‐sum test, differences in distributions were determined using the Kolmogorov–Smirnov (KS) test, and differences in proportions were determined using the chi‐squared test. Statistical testing was conducted in MATLAB. Unless otherwise noted, all reported *p*‐values are from both Wilcoxon and KS tests.

Following Cornblath et al.,^50^ we assessed whether individual CCEP waveforms significantly increased or decreased in amplitude across the 30‐s 1‐Hz stimulation train. We conducted Spearman correlation between the individual CCEP amplitudes and their pulse count.^1^, ^2^, ^3^, ^4^, ^5^, ^6^, ^7^, ^8^, ^9^, ^10^, ^11^, ^12^, ^13^, ^14^, ^15^, ^16^, ^17^, ^18^, ^19^, ^20^, ^21^, ^22^, ^23^, ^24^, ^25^, ^26^, ^27^, ^28^, ^29^, ^30^ For this analysis, on an individual‐level *p*‐values were Bonferroni corrected using each individual's number of CCEP trains being compared, and the corrected *p*‐value was used to determine significance.

To account for individual variability and determine the effect of stimulation frequency and electrode location, we used a linear mixed effects (LME) model (R version 4.3.3). All models included subject ID as a random effect. The following categorical variables were included: subject ID, stimulation frequency (high or low), and electrode location (within or outside of the MTR or SOZ).

All statistical testing was conducted with an alpha level of .05 to determine significance.

## 3 RESULTS

Nineteen individuals began the stimulation paradigm; two did not complete it due to convulsive seizures during the first half of the study. Seventeen participants completed the full protocol (Tables 1 and S1). Nine stimulation sites across five individuals were excluded from analysis due to two seizures and five protocol deviations (human error selecting stimulation parameters or foregoing retesting due to seizures or unpleasant sensations). Of the 17 who completed the protocol, six were adults, all with temporal epilepsy. The other 11 were children, eight with multifocal/diffuse and three with focal epilepsy. No effect of patient age was observed (*p* > .05; Figure S1). Four subjects became seizure‐free in the weeks to months prior to their invasive EEG study; three of these subjects did not have spontaneous seizures for the duration of their clinical monitoring, and one other subject with several seizures each week did not have spontaneous seizures for the duration of clinical monitoring (Table 1). No patients had seizures in the 12 h prior to study participation.

**TABLE 1:** Clinical characteristics.

ID	Age, years	Sex	Etiology	Imaging findings	Seizure distribution	sEEG SOZ	ASMs	Held ASMs	Baseline seizure frequency
1	3	M	Unknown	Normal MRI	Unknown	No spontaneous seizures	None	LCM, TPM, CZP, LEV	Controlled on medication
2	3	F	Structural	Hypothalamic hamartoma	Focal	Hypothalamic hamartoma, RA, RH	None	LCM, CZP	Controlled on medication
3	5	M	Structural	R MTS; L FCD	Unknown	No spontaneous seizures	None	CLB, LCM, VPA	Controlled on medication
4	6	F	Structural, Genetic (TS)	Bilateral cortical tubers, s/p R frontal laser ablation	Focal	RFI	CBD 120 mg BID, FBM 400 mg BID, everolimus 3 mg daily, VGB 250 mg/375 mg		Daily
5	8	M	Unknown (autism)	Transient T2 flare hyperintensity in RH, PET RT hypometabolism	Unknown	No spontaneous seizures	None	CLB, LCM	Weekly to daily
6	8	M	Genetic (*SCN8A* VUS)	Normal MRI	Focal	No spontaneous seizures	None	OXC, CLB	Weekly, then stopped with clobazam
7	12	F	Structural, genetic (TS, LGS)	Multifocal cortical tubers	Multifocal/diffuse (bilateral)	Tonic seizures in LH & LFT, or RH & RFT; diffuse epileptic spasms	CBD 350 mg BID, CLB 15 mg BID, FBM 400 mg TID, LCM 300 mg BID, LEV 1000 mg BID		Multiple daily
8	12	M	Unknown (autism)	LT heterotopia	Multifocal/diffuse (bilateral)	RF, LTI, RA, RH, LFT	CLB 10 mg BID	LCM	3–5 per week
9	14	M	Structural	RF astrocytoma s/p resection, diffuse band heterotopia	Multifocal/diffuse (bilateral)	LP and/or RP with rapid bilateral spread	LCM 150 mg BID	CBD, LMG	Monthly to daily
10	17	M	Structural	Diffuse subcortical band heterotopia	Multifocal/diffuse (bilateral)	LFI with spread to RFI; R hemisphere; diffuse	None	CLB, LCM, TPM	2–4 per month
11	17	M	Unknown (LGS)	Normal MRI	Multifocal/diffuse (bilateral)	Diffuse RF, diffuse LF, LH	None	BRV, LCM, PHB	Daily
12	25	F	Unknown	Normal MRI	Focal	LT superior gyrus, LH, LA	BRV 100 mg BID, LCM 200 mg BID		Weekly
13	26	M	Unknown	RF heterotopia	Focal	LA, LH	ZNS 100 QHS, CLB 10 mg BID, BRV 100 mg BID, LCM 100 mg BID		1 per month
14	26	M	Structural (FCD)	Subtle LT cortical thickening	Focal	LH	VPA ER 1000/1250, LCM 100 mg BID, LMG 100 mg BID		1 per month
15	28	M	Structural (MTS)	L MTS	Focal	LA, LT	CNB 400 mg QHS, CLB 5 mg twice weekly		1 per month
16	29	M	Structural (temporal encephalocele)	LT encephalocele	Focal	LH	LEV 1500 mg BID, LCM 200 mg BID		1–2 per month
17	62	M	Structural (TBI)	Small susceptibility artifact in R postcentral gyrus	Focal	RA, RH	LEV 750 mg, LMG 400 mg BID, OXC 600 mg BID		4 per year

Abbreviations: ASM, antiseizure medication; BID, twice daily; BRV, brivaracetam; CBD, cannabidiol; CLB, clobazam; CNB, cenobamate; CZP, clonazepam; F, female; FBM, felbamate; FCD, focal cortical dysplasia; L, left; LA, left amygdala; LCM, lacosamide; LEV, levetiracetam; LF, left frontal; LFI, left frontal insula; LFT, left frontal temporal; LGS, Lennox–Gastaut syndrome; LH, left hippocampus; LMG, lamotrigine; LP, left parietal; LT, left temporal; LTI, left temporal insula; M, male; MRI, magnetic resonance imaging; MTS, mesial temporal sclerosis; OXC, oxcarbazepine; PET, positron emission tomography; PHB, phenobarbital; QHS, nightly; R, right; RA, right amygdala; RF, right frontal; RFI, right frontal insula; RFT, right frontal temporal; RH, right hippocampus; RP, right parietal; RT, right temporal; s/p, superior/posterior; sEEG, stereo‐electroencephalography; SOZ, seizure onset zone; TBI, traumatic brain injury; TID, three times daily; TPM, topiramate; TS, tuberous sclerosis; VGB, vigabatrin; VPA, valproic acid; VUS, variant of unknown significance; ZNS, zonisamide.

As described in Materials and Methods, each site received a 5‐s “treatment” stimulation train at either high (100 Hz) or low (7 Hz) frequency (Figure 1A). Treatment trains were delivered at .5–4 mA. In each participant, all recording sites (range = 48–122, median = 85) were assessed for CCEPs, yielding 20 828 distinct CCEP‐treatment‐CCEP recordings. A total of 2560 CCEPs were analyzed (see Table S1 for each individual's CCEP counts and Figure S2 for anatomical locations of all detected CCEPs). CCEPs were always present or absent in a given recording electrode for both pretreatment trains (present for 605 stimulation–recording electrode pairs, absent for 8575 stimulation–recording electrode pairs). Furthermore, Spearman test showed strong correlation between CCEP amplitude for both averaged pretreatment trains (ρ = .83, *p* = .0), suggesting that CCEPs were repeatable.

### 3.1 A single 5‐s HFS or LFS train differentially modulates CCEP amplitude

**FIGURE 2:**
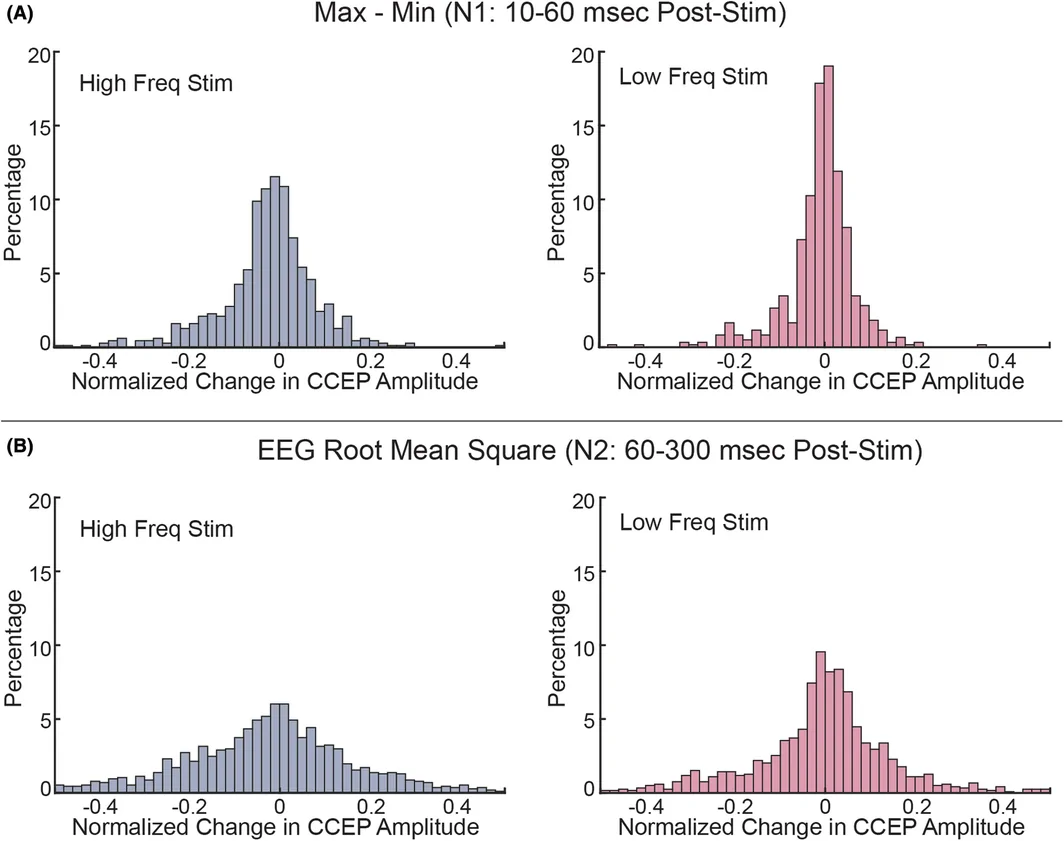
Stimulation‐induced change in corticocortical evoked potential (CCEP) amplitude is frequency‐dependent, independent of quantification method. High‐frequency stimulation (100 Hz, blue) causes a greater reduction in CCEP amplitude than low‐frequency stimulation (7 Hz, pink), regardless of the method used to measure change in CCEP amplitude (*n* = 605 CCEP pairs per frequency; Wilcoxon signed‐rank and Kolmogorov–Smirnov tests, *p* < .01 for N1 measures, *p* < .02 for N2 measures). Methods tested include (A) difference between the maximum and minimum values 10–60 ms poststimulation and (B) the root mean square of the electroencephalographic (EEG) trace 60–300 ms poststimulation. EEG line length and root mean square 10–60 ms poststimulation and EEG line length 60–300 ms poststimulation were also tested; see Figure S3.

To evaluate whether CCEP amplitude could potentially serve as a biomarker of neurostimulation effect, we compared change in CCEP amplitude following single 5‐s LFS and HFS trains. HFS was more likely to reduce CCEP amplitude than LFS (−1.6% vs. −.02%, Wilcoxon signed‐rank test, *p* = .002; KS test *p* = 5.3 × 10^−8^; Figure 2A). To account for interindividual variability, we constructed an LME model with subject ID as a random effect. Stimulation frequency significantly modulated CCEP amplitude, with HFS associated with a 1.7% greater reduction than LFS (95% confidence interval [CI] = −.7% to −2.8%; Table S3). Both nonparametric tests and LME modeling showed that HFS led to significantly greater CCEP amplitude reduction than LFS across all quantification methods (Figures 2 and S3, Table S3).

### 3.2 Epileptiform activity does not account for HFS‐induced CCEP amplitude reductions

We next investigated whether epileptiform activity contributed to the observed CCEP amplitude reductions following HFS. In 14 of 17 individuals, 5‐s treatment trains induced afterdischarges, electrographic seizures, or on two occasions clinical seizures, lasting from 2 to 60 s (Table S2). HFS was significantly more likely to provoke epileptiform activity than LFS (24 of 118 HFS trains vs. 1 of 118 LFS trains, chi‐squared proportion test, *p* = 1.1 × 10^−6^).

**FIGURE 3:**
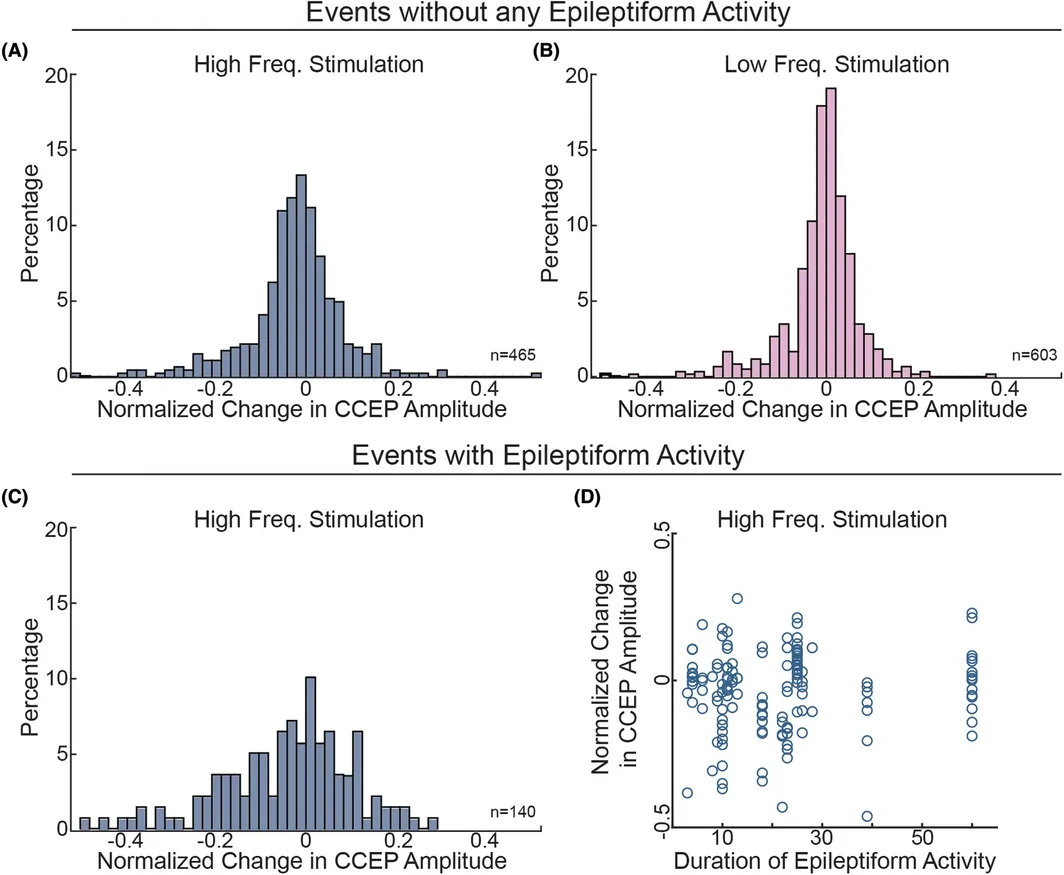
High‐frequency stimulation (HFS) induces a greater reduction in corticocortical evoked potential (CCEP) amplitude, independent of epileptiform activity. (A, B) When stimulation events associated with epileptiform activity are excluded, HFS still results in a greater reduction in CCEP amplitude than low‐frequency stimulation (Wilcoxon rank‐sum and Kolmogorov–Smirnov [KS] tests; *p* < .01). (C) HFS that evoked epileptiform activity produced a similar median change in CCEP amplitude compared to HFS without epileptiform activity (Wilcoxon rank‐sum test, *p* > .05) but significantly altered the distribution of CCEP amplitude (A vs. C, KS test, *p* < .05). (D) Change in CCEP amplitude following HFS‐evoked epileptiform activity did not correlate with the duration of stimulation‐induced epileptiform activity (duration range = 1–60 s, Spearman correlation, *p* = .31, ρ = .09).

To assess the impact of epileptiform activity on CCEP modulation, we compared change in CCEP amplitude following HFS that was or was not followed by epileptiform activity (140 vs. 465 CCEP pairs). Although the distributions differed in shape (KS test, *p* = .004), the median change in CCEP amplitude did not differ significantly (Wilcoxon rank‐sum test, *p* = .60; Figure 3A–C). Additionally, the duration of epileptiform activity did not correlate with the degree of change in CCEP amplitude (Spearman ρ = .09, *p* = .31; Figure 3D). Furthermore, after repeating the overall analysis after excluding all pre‐/posttreatment CCEPs where the treatment train was followed by epileptiform activity, HFS remained more likely than LFS to reduce CCEP amplitude (*n* = 1068 CCEP pairs; *p* < 1.0 × 10^−3^; Figure 3A,B). LME modeling of this subset also predicted that HFS would decrease CCEP amplitude by 1.3% more than LFS (95% CI = −2.4% to −.26%; Table S4). Thus, we conclude that HFS‐induced reductions in CCEP amplitude occur independently of epileptiform activity.

### 3.3 HFS decreases CCEP amplitude independent of total charge flux

We next asked whether the greater total movement of charge (or flux) delivered by HFS, rather than frequency itself, accounted for the observed reductions in CCEP amplitude. To test this, we grouped treatment trains by total charge flux. Stimulation current ranged from .5 to 4 mA, yielding a total charge flux between .0028 and .022 Culombs per train (C/train) for LFS, and between .040 to .32 C/train for HFS. Given fewer CCEP pairs at the lower charge levels, we grouped treatment stimulation trains into two categories: .5–2 mA and 3–4 mA for each stimulation frequency.

**FIGURE 4:**
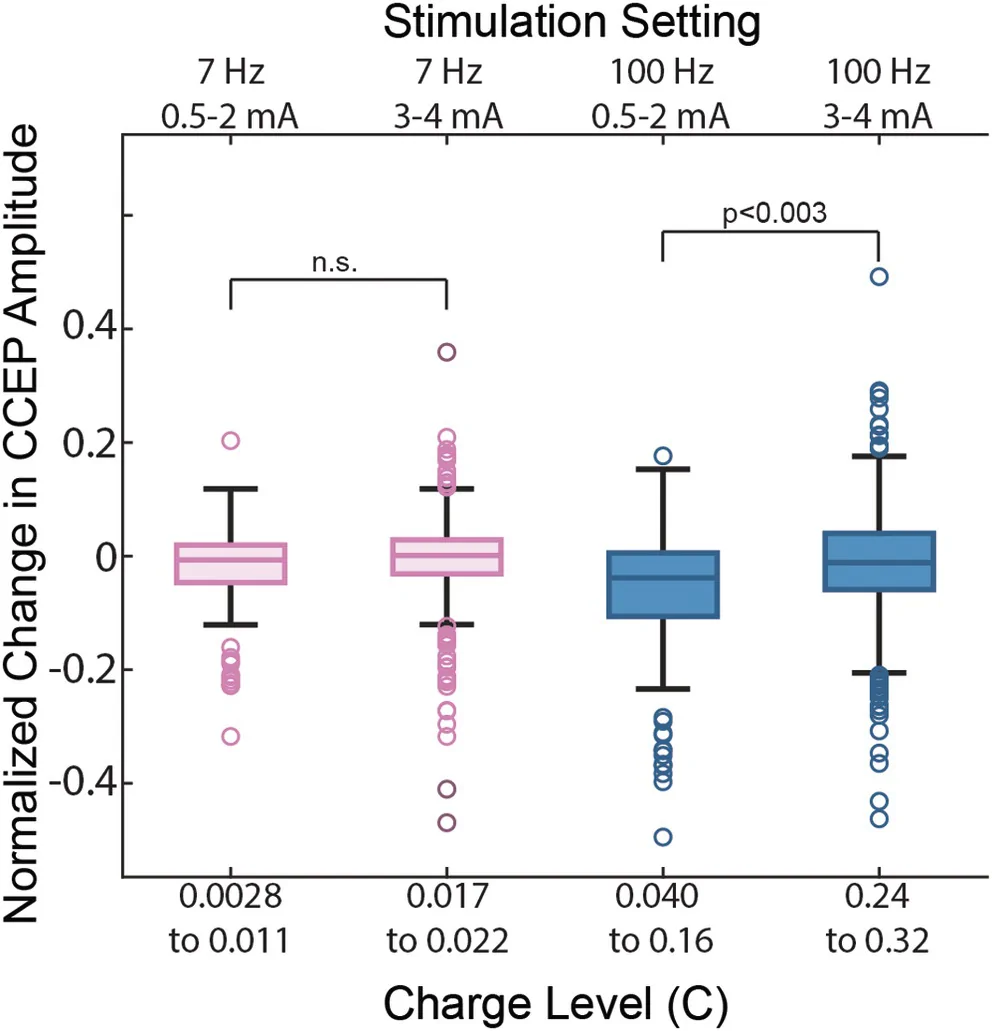
The maximum charge delivered does not correspond to the greatest changes in corticocortical evoked potential (CCEP) amplitude. For low‐frequency stimulation (pink, left), there is no difference between the lower (.0028 to .011 Culombs per train; *n* = 142 CCEP pairs) and higher (.017 to .022 C/train; *n* = 463 CCEP pairs) charge levels (Wilcoxon rank‐sum and Kolmogorov–Smirnov [KS] tests; *p* > .05). For high‐frequency stimulation (blue, right), lower charge (.040 to .16 C/train; *n* = 154 CCEP pairs) results in a greater reduction in CCEP amplitude than higher charge (.24 to .32 C/train; *n* = 451 CCEP pairs; Wilcoxon rank‐sum and KS tests, *p* < .003).

With LFS, no effect of charge flux on CCEP amplitude was observed (Figure 4). However, with HFS, we observed that greater charge flux was associated with a smaller reduction in CCEP amplitude (*p* < .003; Figure 4). This unexpected result could suggest that change in CCEP amplitude does not scale linearly with total charge and that stimulation frequency modulates CCEP amplitude independently of charge flux. Alternatively, the smaller reductions at higher charges may reflect site selection bias, as five subjects had treatment trains with reduced current (1–2 mA vs. 4 mA) only at sensitive sites (e.g., the SOZ or MTR). Regardless, the data indicate that greater charge flux with HFS trains did not cause larger CCEP reductions, suggesting that total charge flux alone does not explain the effect of HFS on CCEP amplitude.

### 3.4 Effects of stimulation on CCEP amplitude are stable across multiple time scales

We next examined the duration of stimulation‐induced changes in CCEP amplitude. First, we assessed whether these changes persisted through the posttreatment CCEP train. Using the approach of Cornblath et al.,^50^ we tested for correlation between an individual CCEP's amplitude and its pulse number within the train. CCEP amplitude was significantly correlated with pulse number in a similar proportion of pre‐ and posttreatment trains (133 vs. 132 of 1210), with a similar proportion of positive correlations (increasing CCEP amplitude) in pre‐ and posttreatment trains (97 of 133 vs. 87 of 132 significant correlations), suggesting that the effect of the treatment stimulation did not diminish over the 30‐s posttreatment CCEP train.

To evaluate longer term effects, we tested whether the order of treatment influenced change in CCEP amplitude. Each electrode received both HFS and LFS in randomized order during the study session (mean session duration = 75.6 ± 2.4 min, intertreatment interval = 6.5–86.5 min, mean = 34.8 min). LFS was more likely to decrease CCEP amplitude when it followed HFS (*p* < .01; Figure S4), suggesting a lasting effect of HFS that occurred minutes to an hour before, whereas HFS effect was not affected by order of stimulation. Moreover, when LFS followed HFS, shorter time intervals between treatment trains were associated with greater reductions in CCEP amplitude (Spearman *ρ* = .2, *p* = 6.2 × 10^−5^; Figure S5A). Neither HFS‐ or LFS‐induced changes in CCEP amplitude were correlated with session duration (Figure S5C,D). Together, these findings suggest that the effect of a single 5‐s HFS train persists on the order of minutes to an hour.

### 3.5 HFS in the SOZ results in greater changes in CCEP amplitude

Previous studies have reported that CCEP amplitude is higher in the SOZ.^25^, ^26^, ^27^, ^28^ We therefore asked whether stimulation‐induced changes in CCEPs would also be amplified in the SOZ. Seven of nine individuals with well‐defined SOZs had seizure onset in the MTR. We observed no difference in pretreatment CCEP amplitude with stimulation within or outside the SOZ (Figure S6; Wilcoxon rank‐sum and KS test, *p* > .1). However, HFS within the SOZ caused a greater reduction in CCEP amplitude compared to HFS outside the SOZ (*p* < .007, 93 vs. 214 CCEP pairs; Figure S7D). LME modeling also predicted that HFS led to larger reduction in N1 CCEP amplitude in the SOZ, whereas N2 CCEP amplitude was less clearly affected by the SOZ (Table S6). In contrast, HFS within and outside of the MTR did not cause a significant change in CCEP amplitude (Figure S7B). Instead, LFS lead to a greater reduction in CCEP amplitude inside the MTR (*p* < 1 × 10^−4^; Figure S7A).

**FIGURE 5:**
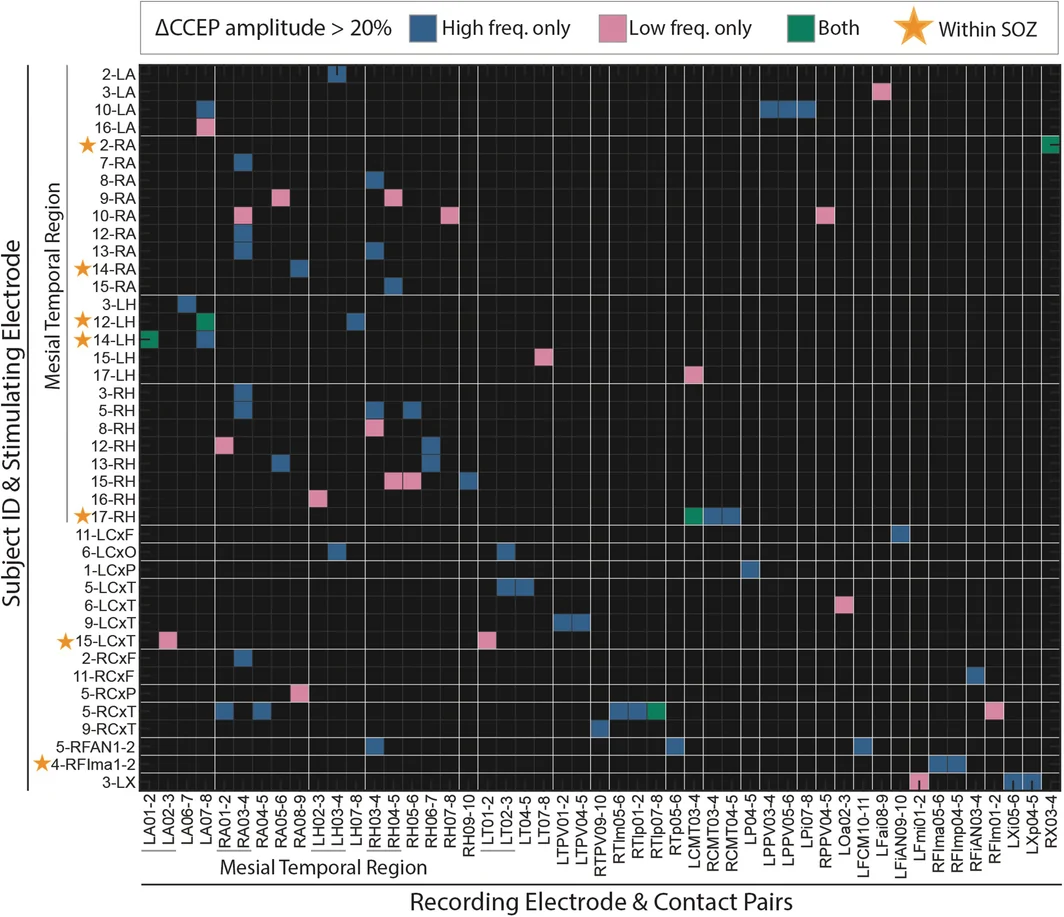
The majority of large changes in corticocortical evoked potential (CCEP) amplitude are seen in the mesial temporal regions. Each colored squared indicates a stimulation–recording electrode pair where high‐frequency stimulation (blue), low frequency stimulation (pink), or both (green) induced a change in CCEP amplitude greater than 20%. Stimulating electrodes are represented as rows, labeled with subject ID and electrode name; L/RA is located in left/right amygdala, L/RH in left/right hippocampus, and L/R CxT/F/P in left/right temporal/frontal/parietal cortex. Recording sites are represented as columns, labeled with electrode name from the invasive electroencephalographic study and the contact pair at which the CCEP was detected. Recording sites may reflect a contact pair in one or several individuals. Yellow stars indicate that stimulating electrode was within a well‐defined focal seizure onset zone (SOZ). Stimulating electrodes are represented as rows, labeled with subject ID and electrode name; L/RA is in left/right amygdala, L/RH in left/right hippocampus, and L/RCxT/F/P is in left/right temporal/frontal/parietal cortex. Recording sites are represented as columns, labeled with electrode name from the invasive electroencephalographic study and the contact pair at which the CCEP was detected.

The anatomical distribution of CCEPs showing posttreatment change greater than 20% is shown in Figure 5. Of the 77 CCEPs with the largest changes, 47 (61%) were stimulated within the MTR, suggesting that this region is intrinsically more excitable. This heightened excitability may explain the larger effect of LFS in this region, or it may reflect other neuroanatomic properties that make the MTR particularly sensitive to LFS. Although these findings support frequency‐specific effects of stimulation in the SOZ and MTR, further data are required to disentangle underlying mechanisms and clinical implications (Figure S8).

## 4 DISCUSSION

To our knowledge, this is the first prospective, randomized comparison of the effect of a single HFS (100 Hz) and LFS (7 Hz) stimulation train on CCEP amplitude. We demonstrated that a single 5‐s stimulation train is sufficient to modulate CCEP amplitude in a frequency‐dependent manner; HFS was more likely than LFS to decrease CCEP amplitude. This effect was consistent across five quantification methods, independent of total charge and evoked epileptiform activity, and persisted for minutes to more than an hour. This change in CCEP amplitude was primarily a shift in distribution (amplitudes both increased and decreased following stimulation) but was also directional (decreased amplitude with HFS). Moreover, we observed region‐specific effects; HFS produced greater reductions in the SOZ, whereas LFS has a stronger effect in the MTR. These findings suggest that CCEP amplitude could serve as an immediate biomarker of neurostimulation effect.

Neurostimulation therapies likely affect multiple neuronal mechanisms operating on time scales from seconds to weeks. CCEP amplitude reflects the magnitude of propagating waves of neuronal depolarization, and may be regarded as an indicator of effective connectivity^32^, ^33^, ^34^, ^35^ and network excitability.^51^, ^52^ It is plausible that treatment stimulation trains activate short time‐scale network mechanisms that decrease neuronal excitability—such as synaptic depression, spike‐frequency adaptation, or γ‐aminobutyric acid type B (GABA_B_)‐mediated inhibition—thereby decreasing the probability of firing in subsequent propagating waves of depolarization and reducing CCEP amplitude. Some of these mechanisms, particularly synaptic depression or GABA_B_ activation, may be preferentially triggered by HFS, potentially explaining the more pronounced amplitude reduction with HFS. However, these short‐term mechanisms are unlikely to account for effects persisting minutes to an hour. This raises the possibility that longer lasting mechanisms—such as synaptic scaling^53^ or regulation of intrinsic excitability^54^—may also be involved.

We observed that the effect of a single train of neurostimulation on CCEP amplitude varied by location; although stimulation caused group‐level decrease in CCEP amplitude, at some sites it caused increased amplitude (e.g., in Figure 2, all bars to the right of the y‐axis represent CCEPs that increased following stimulation), an effect that has been seen in other studies.^55^ Our most robust observation was that HFS and LFS trains differentially changed the distribution of CCEP amplitude across all sites. Treatment stimulation trains depolarize both excitatory and inhibitory neurons, so it may be that the direction of change in CCEP amplitude depends on the extent to which each population is activated.^56^ For example, preferential stimulation of inhibitory populations could decrease inhibition, increasing the number of neurons participating in propagating depolarization, and thereby increase CCEP amplitude. Additionally, local fluctuations in network excitability may change response to short stimulation trains,^50^ and increasing distance between stimulating and recording electrodes likely reduces the magnitude of the observed change in amplitude.^37^, ^41^ Thus, although it is clear that change in CCEP amplitude reflects frequency‐dependent effects of neurostimulation, a finding supported by other recent studies,^43^, ^55^ its long‐term reproducibility at a given location over time and correlation to clinical outcomes with neurostimulation have yet to be established. It may be that other specific features of CCEPs (latency, velocity, etc.), network level analysis of CCEPs, or other passive EEG measures could also prove effective for this purpose.

In this study, we found that decreased CCEP amplitude was associated with factors often linked to epileptiform activity: the SOZ, the MTR, and HFS. Whereas most studies report increased CCEP amplitude^25^, ^26^, ^27^, ^28^ in the SOZ, others have reported decreased CCEP amplitude.^27^, ^50^ In our dataset, pretreatment CCEP amplitude did not significantly differ between stimulation within and outside the SOZ, raising the possibility that change in CCEP amplitude following stimulation may be a more specific biomarker of the SOZ than absolute amplitude. Furthermore, it is interesting that LFS rather than HFS led to an overall greater reduction in CCEP amplitude in the MTR. Given that most focal SOZs were in the MTR (seven of nine patients), this raises the possibility that differential response to LFS versus HFS could help determine whether one or both hippocampi are the source of a patient's habitual seizures. However, a larger dataset is necessary to clarify the relationships among the SOZ, MTRs, HFS, and LFS.

This study has several limitations. First, HFS had greater charge flux than LFS, making charge flux a potential confounding variable. However, the finding that increased charge flux did not reduce CCEP amplitude within either frequency group provides assurance that charge alone is not the cause of the observed effects. Second, although we used LME modeling to account for individual differences, most patients did not have enough CCEPs to power individual‐level comparisons of stimulation frequency effects. Future studies should increase the number of CCEPs recorded in each patient, potentially by first identifying sites that generate CCEPs and then applying stimulation paradigms at those locations. Third, no control paradigm with pre‐ and posttreatment CCEP trains and no intervening treatment train were performed. Previous literature suggests that CCEPs themselves modulate cortical networks and epileptiform activity,^38^, ^57^, ^58^ and that CCEPs may be variable within 30‐s trains or across trials.^38^, ^41^ Thus, although this randomized comparison established that HFS has a differential effect of CCEP amplitude compared to LFS, we cannot quantify the effects of the pretreatment CCEP stimulation train on posttreatment CCEPS, nor can we compare baseline CCEP variability between pre‐ and posttreatment trains. Finally, each individual only underwent one stimulation session. To fully evaluate the utility of stimulation‐induced changes in CCEP amplitude as a clinical biomarker, it will be important to establish the consistency of site‐specific effects over multiple sessions spanning days.

## 5 CONCLUSIONS

The goal of this study was to establish potential biomarkers of immediate response to neurostimulation, to be used in data‐driven algorithms that could be used to rapidly optimize a patient's neurostimulation device. We present compelling evidence that a single 5‐s train of neurostimulation has an immediate effect on CCEP amplitude. To further determine the utility of change in CCEP amplitude as a biomarker for clinical parameter optimization, results could be validated in patients across multiple sessions, computer modeling studies could provide additional support for the mechanisms proposed in this study, and long‐term clinical studies could correlate this measure with long‐term clinical outcomes. Together, these efforts will advance the development of personalized neurostimulation strategies that improve seizure control and quality of life for patients with epilepsy.

## AUTHOR CONTRIBUTIONS

**Bethany J. Stieve:** Conceptualization; methodology; software; investigation; data curation; formal analysis; writing—original draft preparation; review and editing. **Aaron F. Struck:** Conceptualization; methodology; investigation; writing—review and editing. **Dillon Scott:** Methodology; data curation; writing—review and editing. **Richard Chappell:** Formal analysis; writing—review and editing. **Andrew T. Knox:** Conceptualization; methodology; investigation; formal analysis; writing—review and editing; supervision.

## CONFLICT OF INTEREST STATEMENT

None of the authors has any conflict of interest to disclose. We confirm that we have read the Journal's position on issues involved in ethical publication and affirm that this report is consistent with those guidelines.

## Supporting information


**Figure S1.** Stimulation‐induced changes in corticocortical evoked potential (CCEP) amplitude do not vary with age. (A) Change in amplitude of 774 CCEP pairs recorded from 11 pediatric participants. (B) Change in amplitude of 436 CCEP pairs recorded from six adult participants. No significant differences were observed between the distributions (Wilcoxon rank‐sum and Kolmogorov–Smirnov tests, *p* > .05), whether analyzing CCEP amplitudes following all stimulation (A, B) or stratifying by stimulation frequency (data not shown).
**Figure S2.** Detected corticocortical evoked potentials (CCEPs) across 17 individuals. Each colored squared indicates a stimulation and recording electrode pair where a CCEP was detected. Stimulating electrodes are represented as rows, labeled with subject ID and electrode name; L/RA is in left/right amygdala, L/RH in left/right hippocampus, and L/RCxT/F/P is in left/right temporal/frontal/parietal cortex, with the remaining rows in other cortical locations. Recording sites are represented as columns, labeled with electrode name from the invasive electroencephalographic study and the contact pair at which the CCEP was detected. Recording sites may reflect a contact pair in one or several individuals.
**Figure S3.** Stimulation‐induced change in CCEP amplitude is frequency‐dependent, independent of quantification method. High‐frequency (100 Hz, blue) stimulation causes a greater reduction in CCEP amplitude than low‐frequency (7 Hz, pink) stimulation, regardless of the method used to measure change in CCEP amplitude (*n* = 605 CCEP pairs per frequency; Wilcoxon signed‐rank and Kolmogorov–Smirnov tests, *p* < .01 for N1 measures, *p* < .02 for N2 measures). Methods tested include (A) electroencephalographic (EEG) root mean square 10–60 ms poststimulation, (B) EEG line length 10–60 ms poststimulation, and (C) EEG line length 60–300 ms poststimulation. The difference between the maximum and minimum values 10–60 ms poststimulation and the root mean square of the EEG trace 60–300 ms poststimulation were also tested; see Figure 2.
**Figure S4.** Order of stimulation influences change in corticocortical evoked potential (CCEP) amplitude. (A, B) Change in CCEP amplitude when the first stimulation at the electrode site is low‐frequency stimulation (A; *n* = 323 CCEP pairs) is significantly different than at electrode sites where low‐frequency stimulation occurs after high frequency (B; *n* = 282 CCEP pairs; Wilcoxon rank‐sum and Kolmogorov–Smirnov tests, *p* < .01). (C, D) There is no difference in change in CCEP amplitude whether high‐frequency stimulation at an electrode site is before (C; *n* = 323 CCEP pairs) or after (D; *n* = 282 CCEP pairs) low‐frequency stimulation.
**Figure S5.** Stimulation‐induced changes in corticocortical evoked potential (CCEP) amplitude are persistent. (A) When low‐frequency stimulation is after high‐frequency stimulation at an electrode site, there is a significant correlation between time since high‐frequency stimulation and change in CCEP amplitude (Spearman correlation, ρ = .2, *p* = 6.2 × 10^−5^), indicating that CCEP amplitude is more likely to be decreased when less time has passed since high‐frequency stimulation at the same site. (B) When high‐frequency stimulation is delivered after low‐frequency stimulation, there is no correlation between the time interval and change in CCEP amplitude (Spearman correlation, ρ = −.03, *p* = .6). (C, D) Change in CCEP amplitude does not correlate with the time since the study session started, for either low‐frequency stimulation (C; Spearman correlation, ρ = −.01, *p* = .8) or high‐frequency stimulation (*D*; ρ = −.06, *p* = .1).
**Figure S6.** Pretreatment corticocortical evoked potential (CCEP) amplitude does not differ with stimulation in the seizure onset zone. Average peak‐to‐peak amplitude of CCEPs is shown from stimulation outside of (A; median = 107.0 μV) and within (B; median = 93.4 μV) the seizure onset zone. No significant difference was observed between the distributions (Wilcoxon rank‐sum and Kolmogorov–Smirnov tests, *p* > .1).
**Figure S7.** Stimulation in the seizure onset zone and the mesial temporal region may decrease corticocortical evoked potential (CCEP) amplitude. (A, B) Change in CCEP amplitude following low‐frequency stimulation (7 Hz, pink) outside of (i) or within (ii) the mesial temporal region (MTR; A; *n* = 17 individuals) and seizure onset zone (SOZ; B; *n* = 9 individuals). The number of CCEP pairs (*n*) and the median change in CCEP amplitude (center value) are noted for each distribution. Low‐frequency stimulation within the MTR led to a greater reduction in CCEP amplitude compared to outside the MTR (Ai vs. Aii; *p* < 1 × 10^−4^). In contrast, CCEP amplitude was not differentially affected by low‐frequency stimulation outside of versus within the SOZ (Bi vs. Bii; *p* > .05). (C, D) Changes in CCEP amplitude following high‐frequency stimulation (100 Hz, blue), comparing stimulation outside (i) and within (ii) the MTR (C) and SOZ (*D*). CCEP amplitude was not differentially affected by high‐frequency stimulation outside of versus within the MTR (Ci vs. Cii; *p* > .05). In contrast, high‐frequency stimulation within the SOZ led to greater reduction in CCEP amplitude compared to outside the SOZ (Di vs. Dii; *p* < .007). All reported *p*‐values are from conducting both Wilcoxon rank‐sum and Kolmogorov–Smirnov tests.
**Figure S8.** Changes in corticocortical evoked potential (CCEP) amplitude following stimulation in the seizure onset zone (SOZ) and mesial temporal region (MTR). (A, B) Change in CCEP amplitude following low‐frequency (A) or high‐frequency (B) stimulation (1) within the MTR and outside of the SOZ (Ai, Bi), (2) within the SOZ and outside of the MTR (Aii, Bii), and (3) within both the MTR and SOZ (Aiii, Biii), across the nine individuals included in the SOZ analysis (see Table S4). The number of CCEP pairs (*n*) and the median of the distribution (centered) are displayed for each distribution.

**Table S1.** Subject‐specific stimulation results.
**Table S2.** Epileptiform activity.
**Table S3.** Results of linear mixed effect modeling across quantification methods of corticocortical evoked potential amplitudes.
**Table S4.** Linear mixed effect model‐predicted change in corticocortical evoked potential amplitude with high‐frequency stimulation that is not followed by epileptiform activity.
**Table S5.** Well‐defined seizure onset zones.
**Table S6.** Linear mixed effect model‐predicted change in corticocortical evoked potential amplitude with high‐frequency stimulation for electrodes within the seizure onset zone.
**Table S7.** Distribution of corticocortical evoked potentials with the largest stimulation‐induced change in amplitude.
**Table S8.** Epilepsy surgery outcomes.

## Data Availability

The data that support the findings of this study are available on request from the corresponding author. The data are not publicly available due to privacy or ethical restrictions.
