# Supplementary material for: Corticocortical evoked potential amplitude is altered by cortical stimulation in epilepsy
Source: Epilepsia. 2026 May 19;67(8):4219–33. doi: 10.1002/epi.70289 (PMC13525592; doi:10.1002/epi.70289)
Supplement: Supplementary file 1 [file EPI-67-4219-s001.docx]

**Supplementary Table 1. Subject-Specific Stimulation Results**

| Subject ID | # Stimulation Sites | Reason for <8 Stimulation Sites | # Recording Sites | # CCEPs | # Electrographic Events |
| --- | --- | --- | --- | --- | --- |
| 1 | 8 | n/a | 108 | 20 | 1 |
| 2 | 8 | n/a | 90 | 148 | 6 |
| 3 | 8 | n/a | 89 | 284 | 5 |
| 4 | 8 | n/a | 83 | 164 | 1 |
| 5 | 8 | n/a | 122 | 236 | 1 |
| 6 | 5 | Provoked seizure,  protocol deviation | 85 | 196 | 4 |
| 7 | 6 | Protocol deviation | 113 | 36 | 0 |
| 8 | 6 | Protocol deviation | 85 | 76 | 1 |
| 9 | 8 | n/a | 98 | 168 | 3 |
| 10 | 8 | n/a | 101 | 172 | 1 |
| 11 | 8 | n/a | 64 | 220 | 1 |
| 12 | 6 | Protocol deviation | 66 | 244 | 4 |
| 13 | 6 | 6 electrodes implanted | 53 | 92 | 5 |
| 14 | 4 | 7 electrodes implanted, provoked seizure,  protocol deviation | 48 | 112 | 1 |
| 15 | 7 | 7 electrodes implanted | 56 | 132 | 1 |
| 16 | 7 | 7 electrodes implanted | 56 | 136 | 0 |
| 17 | 7 | 7 electrodes implanted | 58 | 124 | 0 |

Stimulation paradigm consists (1) a 30-second 1Hz stimulation train to generate baseline CCEPs, (2) a five second “treatment” stimulation train at either 7Hz or 100Hz, and (3) a second 30-second 1Hz stimulation train to generate post-treatment CCEPs. Electrographic events are runs of afterdischarges, electrographic seizures, or electroclinical seizures that immediately followed the five-second treatment train (see Supplementary Table 2).

**Supplementary Table 2. Epileptiform Activity**

| Subject ID | Stimulation Site | Stimulation Site within SOZ | Stimulation Frequency (Hz) | Stimulation Current Amplitude (mA) | Duration of epileptiform activity (s) | Max Frequency | Spread | Clinical Correlate | Time from cessation of epileptiform activity to CCEP train (s) |
| --- | --- | --- | --- | --- | --- | --- | --- | --- | --- |
| 1 | LH 1-2 | n/a | 100 | 4 | 12 | 1 | No | Whines and restless following  Stimulation (20 s) | 25 |
| 2 | LH 1-2 | No | 100 | 2 | 28 | 5 | LA | none | 39 |
| 2 | RA 1-2 | Yes | 7 | 4 | 12 | 0.5 | No | none | 17 |
| 2 | RA 1-2  (pre CCEP train) | Yes | 1 | 8 | 41 | 3 | RH | wakes | 38 to treatment |
| 2 | RA 1-2 | Yes | 100 | 4 | 8 | 2 | No | none | 23 |
| 2 | RFla 4-5 | No | 100 | 4 | 13 | 6 | No | Moans, fidgety movements, desaturation | 32 |
| 2 | LA 1-2 | Yes | 100 | 2 | 3 | 4 | No | None | 25 |
| 3 | LA 1-2 | n/a | 100 | 3 | 12 | 1.5 | L hemi | None | 25 |
| 3 | LH 1-2 | n/a | 100 | 3 | 22 | 9 | LA | Fidgets under blanket (covered), no other change | 40 |
| 3 | FCD 2-3 (LXa2-3) | n/a | 100 | 3 | 23 | 7 | No | none | 29 |
| 3 | RA 1-2 | n/a | 100 | 3 | 60 | 7 | RH | Fidgets under blanket (covered), no other change | 56 |
| 3 | LF 5-6 (LFmi5-6) | n/a | 100 | 3 | 25 | 7 | No | none | 32 |
| 4 | RTIm 3-4 | Yes | 100 | 4 | 4 | 6 | No | None | 32 |
| 5 | LH 1-2 | n/a | 100 | 2 | 26 | 10 | No | None | 30 |
| 6 | LT 5-6 | n/a | 100 | 2 | 18 | 9 | No | Intermittent fidgety movements, not clear | 47 |
| 6 | LOa 5-6 | n/a | 100 | 2 | 28 | 9 | No | none | 20 |
| 6 | LH 2-3  (post CCEP train) | n/a | 1 | 8 | 60 | 4.5 | L TP | O2 desaturation, becomes pale, raises hands and whines for last 10 seconds | n/a |
| 6 | RPa 6-7 | n/a | 100 | 0.5 | 2 |  |  |  |  |
| 8 | RH 1-2 | n/a | 100 | 4 | 10 | 1 | No | none | 34 |
| 9 | LPip 3-4 | Yes | 100 | 4 | 2 | 1 | No | none | 29 |
| 9 | RH 1-2 | No | 100 | 4 | 11 | 1 | RA | none | 21 |
| 9 | RA 1-2 | No | 100 | 4 | 6 | 1 | RH | none | 20 |
| 10 | LA 1-2 | n/a | 100 | 1 | 10 | 10 | LH | none | 48 |
| 11 | LA 1-2 | n/a | 100 | 4 | 60 | 20 | LH, LFia | Intermittent fidgety movements and tries to get out of bed | 32 |
| 12 | LP 5-6 | No | 100 | 4 | 4 | 1 | No | none | 32 |
| 12 | LA 1-2 | Yes | 100 | 4 | 41 | 20 | L hemi | Behavioural arrest, unresponsive | 125 |
| 12 | RA 1-2 | No | 100 | 4 | 25 | 5 | RH | none | 21 |
| 13 | LOF 10-11 | No | 100 | 3 | 3 | 1 | No | none | 24 |
| 13 | LC 10-11 | No | 100 | 3 | 7 | 6 | No | none | 34 |
| 13 | RA 1-2 | No | 100 | 3 | 24 | 6 | RH | When asked, reports a memory of a dream | 33 |
| 13 | RH 2-3 | No | 100 | 3 | 5 | 10 | No | When asked, reports deja vu | 33 |
| 13 | LA 1-2 | No | 100 | 3 | 39 | 20 | LH | When asked, reports feeling nervous | 83 |
| 14 | RH 1-2 | No | 100 | 3 | 9 | 0.5 | No | none | 30 |
| 15 | RH 1-2 | n/a | 100 | 4 | 12 | 0.5 | No | none | 34 |

Electrographic events are runs of afterdischarges, electrographic seizures, or electroclinical seizures. Most immediately followed the five-second train; epiletiform events during CCEP trains were excluded from analysis. R: right; L: left; m: medial; a: anterior; p: posterior; s: superior; i: inferior; X: lesion; A: amygdala; H: hippocampus; F: frontal; : frontal insula; P: parietal; T: temporal; O: occipital; C: cingulate

**Supplemental Table 3. Results of linear mixed effect modeling across quantification methods of CCEP amplitudes.**

| **∆CCEP Amplitude ~ Stimulation Frequency + (1\|SubID)** | | | |
| --- | --- | --- | --- |
| **Quantification Method** | | **Coefficient of Fixed Effects**  **[95% Confidence Interval]** | |
| Time interval post-stim. (msec) | Amp. Calculation | Stimulation Frequency | Intercept |
| 10-60 | Max. – Min. | **-0.017*** [-0.028 to -0.007] | -0.011 [-0.022 to +0.0006] |
| 10-60 | EEG Line Length | **-0.025*** [-0.035 to -0.016] | -0.004 [-0.013 to +0.005] |
| 60-300 | EEG Line Length | **-0.025*** [-0.035 to -0.016] | -0.004 [-0.013 to +0.005] |
| 60-300 | RMS | **-0.026*** [-0.038 to -0.015] | -0.005 [-0.015 to +0.005] |

****P* < 0.05**

**Supplementary Table 4. Linear Mixed Effect Model - Predicted Change in CCEP Amplitude with High Frequency Stimulation that is Not Followed by Epileptiform Activity**

| **∆CCEP Amplitude ~ Stimulation Frequency + (1\|SubID)** | |
| --- | --- |
| **Coefficient of Fixed Effects [95% Confidence Interval]** | |
| High Frequency Stimulation | Intercept |
| **-0.013*** [-0.024 to -0.003] | -0.010 [-0.02 to +0.01] |

Analysis of 1,068 CCEPs without associated epileptiform activity shows that high frequency stimulation is associated with decreased CCEP amplitude independent of evoked epileptiform activity. **P* < 0.05

**Supplementary Table 5. Well-defined Seizure Onset Zones**

| Subject ID | Electrode Contacts Contained in the Seizure Onset Zone | | | | | |
| --- | --- | --- | --- | --- | --- | --- |
| 2 | RX 2-3 | RA 1-2 | RH 1-2 |  |  |  |
| 4 | RFIma 1-3 | RFImp 1-3 | RFIps 5-7 |  |  |  |
| 9 | LPip 1-5 | LPsp 1-6 | RPi 1-6 | RPs 1-5 | RFm 1-4 | RFp 1-5 |
| 12 | LA 1-6 | LH 1-8 | LTsp 1-3 |  |  |  |
| 13 | LA 1-4 | LH 1-3 |  |  |  |  |
| 14 | LH 1-2 |  |  |  |  |  |
| 15 | LTa 3-8 | LA 4-8 |  |  |  |  |
| 16 | LH 1-2 | LH 2-3 | LA 1-2 | LA 2-3 |  |  |
| 17 | RA 1-10 | RH 1-10 |  |  |  |  |

Seizure onset zones (SOZ) for individuals with a consistent, focal onset seizures. The SOZ was defined as the set of all contact pairs that were included in the first five seconds of spontaneous typical clinical seizures recorded during the patient’s invasive EEG study. Individuals with diffuse or multifocal SOZs, as well as individuals who had no spontaneous seizures during invasive EEG monitoring were not included in SOZ analysis. R: right; L: left; m: medial; a: anterior; p: posterior; s: superior; i: inferior; X: lesion (hypothalamic hamartoma); A: amygdala; H: hippocampus; FI: frontal insula; P: parietal; T: temporal

**Supplementary Table 6. Linear Mixed Effect Model – Predicted Change in CCEP Amplitude with High Frequency Stimulation for Electrodes Within the Seizure Onset Zone (SOZ)**

| **∆CCEP Amplitude ~ Stimulating Electrode-SOZ + Recording Electrode-SOZ + (1\|SubID)** | | | | | |
| --- | --- | --- | --- | --- | --- |
| **Quantification Method** | | **Coefficient of Fixed Effects**  **[95% Confidence Interval]** | | | |
| Time interval post-stim. (msec) | Amplitude Calculation | Stimulating Electrode within the SOZ | Recording Electrode within the SOZ | Intercept | |
| 10-60 | Max. – Min. | **-0.045*** [-0.07 to -0.02] | +0.034 [-0.005 to +0.07] | | -0.011 [-0.03 to +0.008] |
| 10-60 | EEG Line Length | **-0.046*** [-0.07 to -0.02] | **+0.048*** [+0.01 to +0.08] | | **-0.021*** [-0.04 to -0.002] |
| 60-300 | EEG Line Length | **-0.022*** [-0.042 to -0.002] | +0.015 [-0.014 to +0.043] | | **-0.024*** [-0.067 to -0.004] |
| 60-300 | Root Mean Squared | +0.009 [-0.014 to +0.031] | **+0.036*** [-0.067 to -0.004] | | **-0.028*** [-0.044 to -0.011] |

****P* < 0.05**

|  | CCEPs | Subjects | Low Frequency Stim. | High Frequency Stim. | First Stimulation at Site | Recording Electrode in SOZ | Stimulating Electrode in SOZ | Recording Electrode in MTR | Stimulating Electrode in MTR |
| --- | --- | --- | --- | --- | --- | --- | --- | --- | --- |
| All CCEPs | 1,210  (100%) | 17  (100%) | 605  (50%) | 605  (50%) | 605  (50%) | 112  (10%) | 292  (24%) | 46  (4%) | 456  (38%) |
| CCEPs with >20% change in amplitude | 77  (6%) | 17  (100%) | 25  (33%) | 52  (68%) | 36  (47%) | 5  (7%) | 16  (21%) | 8  (10%) | 42  (55%) |

**Supplementary Table 7. Distribution of Cortico-Cortical Evoked Potentials (CCEPs) with the Largest Stimulation-induced Change in Amplitude**

**Supplementary Table 8. Epilepsy Surgery outcomes**

| Subject ID | Final SOZ | Surgical Procedure | Outcome (6 months or greater) | Time of outcome (months) |
| --- | --- | --- | --- | --- |
| 1 | No spontaneous seizures | None – controlled on medications | Seizure free for 9 months, then seizure recurrence, ongoing workup | n/a |
| 2 | Hypothalamic Hammartoma, right amygdala and hippocampus | Laser ablation of hypothalamic hamartoma | Seizure free | 13 |
| 3 | No spontaneous seizures | None – controlled on medications | Seizure free | 15 |
| 4 | Right frontal insula | Laser ablation right insula | n/a | n/a |
| 5 | No spontaneous seizures | None – bilateral seizures, behavioural concerns preclude RNS | Daily -> monthly with medication change | 10 |
| 6 | No spontaneous seizures | None – controlled on medications | Seizure free, medications weaned | 25 |
| 7 | Tonic seizures starting at left or right hippocampus with rapid involvement of left or right frontotemporal networks. Also epileptic spasms with diffuse onset | Offered corpus callosotomy vs RNS. After second opion, family is moving forward with RNS | n/a | n/a |
| 8 | Right frontal, left temporal insula, right amygdala/hippocampus, left frontotemporal | VNS | Decreased from 3-5 sz/week to 1 sz/week | 18 |
| 9 | Left parietal and/or right parietal with rapid bilateral spread | RNS bilateral CMN | Improved frequency, but continued daily seizures | 16 |
| 10 | L frontal insula with spread to R, others with diffuse or R hemisphere onset . | RNS bilateral CMN | n/a | n/a |
| 11 | Diffuse right frontal, diffuse left frontal and left hippocampus | RNS bilateral CMN | 80% reduction in seizures and improved behavior | 15 |
| 12 | Left superior temporal gyrus and hippocampus | RNS L hippocampus and superficial temporal strip | Initially improved but now unchanged, auras 2 in 3 days, sporadic GTCs | 25 |
| 13 | Left Amygdala / Hippocampus | RNS (L hippocampus and ANT) | Improved to few yearly | 9 |
| 14 | Left Hippocampus | RNS (L hippocampus and cingulate) | n/a | n/a |
| 15 | Left Amygdala | RNS L hippocampus and superficial temporal strip | No improvement, 2-3 focal seizures weekly | 9 |
| 16 | Left Hippocampus | L temporal encephalocele repair | n/a | n/a |
| 17 | Right Amygdala / Hippocampus | RNS (R hippocampal, CMN) | SUDEP 1 month following RNS placement | n/a |
