# Supplementary material for: Corticocortical evoked potential amplitude is altered by cortical stimulation in epilepsy
Source: Epilepsia. 2026 May 19;67(8):4219–33. doi: 10.1002/epi.70289 (PMC13525592; doi:10.1002/epi.70289)
Supplement: Supplementary file 2 [file EPI-67-4219-s002.docx]

**Supplementary Figure 1. Stimulation-Induced Changes in CCEP Amplitude Do not Vary with Age. (A).** Change in amplitude of 774 CCEP pairs recorded from 11 pediatric participants. **(B)** Change in amplitude of 436 CCEP pairs recorded from six adult participants. No significant differences were observed between the distributions (Wilcoxon rank sum and KS tests, p>0.05), whether analyzing CCEP amplitudes following all stimulation (*A, B*) or stratifying by stimulation frequency (data not shown).

**Supplementary Figure 2. Detected CCEPs Across 17 Individuals.** Each colored squared indicates a stimulation and recording electrode pair where a CCEP was detected. Stimulating electrodes are represented as rows, labeled with subject ID and electrode name; L/RA is in left/right amygdala, L/RH in left/right hippocampus, and L/RCxT/F/P is in left/right temporal/frontal/parietal cortex, with the remaining rows in other cortical locations. Recording sites are represented as columns, labeled with electrode name from the invasive EEG study and the contact pair at which the CCEP was detected. Recording sites may reflect a contact pair in one or several individuals.

**Supplementary Figure 3. Stimulation-induced change in CCEP amplitude is frequency-dependent, independent of quantification method.** High frequency (100 Hz, *blue*) stimulation causes a greater reduction in CCEP amplitude than low frequency (7 Hz, *pink*) stimulation, regardless of the method used to measure change in CCEP amplitude (n=605 CCEP pairs per frequency; Wilcoxon signed rank and KS tests, p<0.01 for N1 measures, p<0.02 for N2 measures). Methods tested include **(A)** EEG root mean square 10-60 msec post-stimulation, **(B)** EEG line length 10-60 msec post-stimulation and **(C)** EEG line length 60-300 msec post-stimulation. The difference between the maximum and minimum values 10-60 milliseconds (msec) post-stimulation and the root mean square of the EEG trace 60-300 msec post-stimulation were also tested, see Figure 2.

**Supplementary Figure 4. Order of Stimulation Influences Change in CCEP Amplitude.**

**(A-B)** Change in CCEP amplitude when the first stimulation at the electrode site was low frequency stimulation (*A*, n=323 CCEP pairs), is significantly different than at electrode sites where low frequency stimulation occurs after high frequency (*B*, n=282 CCEP pairs; Wilcoxon Rank Sum and KS tests, p<0.01). **(C-D)** There is no difference in change in CCEP amplitude whether high frequency stimulation at an electrode site is before (*C*, n=323 CCEP pairs) or after (*D*, n=282 CCEP pairs) low frequency stimulation.

**Supplementary Figure 5. Stimulation-Induced Changes in CCEP Amplitude Are Persistent. (A)** When low frequency stimulation is after high frequency stimulation at an electrode site, there is a significant correlation between time since high frequency stimulation and change in CCEP amplitude (Spearman’s correlation, rho=0.2, p=6.2*10^-5^), indicating that CCEP amplitude is more likely to be decreased when less time has passed since high frequency stimulation at the same site. **(B)** When high frequency stimulation is delivered after low frequency stimulation, there is no correlation between the time interval and change in CCEP amplitude (Spearman’s correlation, rho=-0.03, p=0.6). **(C-D)** Change in CCEP amplitude does not correlate with the time since the study session started, for either low frequency stimulation, (*C*; Spearman’s correlation, rho=-0.01, p=0.8) or high frequency stimulation (*D*; rho=-0.06, p=0.1).

**Supplementary Figure 6. Pre-treatment CCEP Amplitude Does Not Differ with Stimulation in the Seizure Onset Zone.** Average peak-to-peak amplitude of CCEPs from stimulation outside of (*A*, median=107.0 µV) and within (*B*, median=93.4 µV) the seizure onset zone. No significant difference was observed between the distributions (Wilcoxon rank sum and KS tests, p>0.1).

**Supplementary Figure 7. Stimulation in the seizure onset zone and the mesial temporal region may decrease CCEP amplitude. (A-B)** Change in cortico-cortical evoked potential (CCEP) amplitude following low frequency stimulation (7 Hz, *pink*) outside of (*i*) or within (*ii*) the mesial temporal region (MTR, *A*; n=17 individuals) and seizure onset zone (SOZ, *B*; n=9 individuals). The number of CCEP pairs (*n*) and the median change in CCEP amplitude (*center value*) are noted for each distribution. Low-frequency stimulation within the MTR led to a greater reduction in CCEP amplitude compared to outside the MTR (*Ai* vs. *Aii*; p<1*10^-4^). In contrast, CCEP amplitude was not differentially affected by low frequency stimulation outside of versus within the SOZ (*Bi* vs. *Bii*; p>0.05). **(C-D)** Changes in CCEP amplitude following high-frequency stimulation (100 Hz, *blue*), comparing stimulation outside (*i*) and within (*ii*) the MTR (*C*) and SOZ (*D*). CCEP amplitude was not differentially affected by high frequency stimulation outside of versus within the MTR (*Ci* vs. *Cii*; p>0.05). In contrast, high frequency stimulation within the SOZ led to greater reduction in CCEP amplitude compared to outside the SOZ (*Di* vs. *Dii*; p<0.007). All reported p-values are from conducting both Wilcoxon rank sum and KS tests.

**Supplementary Figure 8. Changes in Cortico-Coritcal Evoked Potential (CCEP) Amplitude following Stimulation in the Seizure Onset Zone (SOZ) and Mesial Temporal Region (MTR).** (**A-B**) Change in CCEP amplitude following low-frequency (*A*) or high-frequency (*B*) stimulation 1) within the MTR and outside of the SOZ (*Ai, Bi*) 2) within the SOZ and outside of the MTR (*Aii, Bii*), and 3) within both the MTR and SOZ (*Aiii, Biii*), across the 9 individuals included in the SOZ analysis (see **Supplementary Table 4**). The number of CCEP pairs (*n*) and the median of the distribution (*centered*) are displayed for each distribution.
